# Association between severe acute malnutrition in childhood and hematological disorders in adulthood: the Lwiro follow-up study in the Eastern Democratic Republic of the Congo

**DOI:** 10.1186/s40795-023-00783-0

**Published:** 2023-11-11

**Authors:** Aline Bedha, Tony Shindano, Michel P. Hermans, Violaine Havelange, Samuel Makali, Jimmy Minani, Gaylord Ngaboyeka, Edwige Kunaba, Philippe Donnen, Michelle Dramaix, Ghislain Bisimwa, Pacifique Mwene-Batu

**Affiliations:** 1 École Régionale de Santé Publique, Université Catholique de Bukavu, Bukavu, Democratic Republic of the Congo; 2 Hôpital Provincial General de Reference de Bukavu, Université Catholique de Bukavu, Bukavu, Democratic Republic of the Congo; 3 Division of Endocrinology & Nutrition, Cliniques Universitaires St-Luc, Université Catholique de Louvain, Brussels, Belgium; 4 Services d’hématologie, Cliniques Universitaires St-Luc, Université Catholique de Louvain, Brussels, Belgium; 5 Ecole de Santé Publique, Université Libre de Bruxelles, 1070 Brussels, Belgium; 6 Faculté de Médecine, Université de Kaziba, Kaziba, Democratic Republic of the Congo

**Keywords:** Severe acute malnutrition, Follow-up, DRC, Long-term, Hematological disorders

## Abstract

**Background:**

Despite growing evidence on the short-term deleterious effects of severe acute malnutrition (SAM) in childhood on hematopoiesis, little is known about the long-term hematological effects of SAM in low-income countries (LICs). Our study explored the association between childhood SAM and hematological disorders in adults 11 to 30 years after post-SAM nutritional rehabilitation.

**Methods:**

This follow up study investigated 97 adults (mean age 32 years) treated for SAM during childhood in eastern Democratic Republic of the Congo (DRC) between 1988 and 2007. Participants were compared to 97 aged- and sex-matched adult controls living in the same community with no history of SAM. Outcomes of interest were hematological characteristics and disorders in adulthood, assessed by various biological markers. Logistic and linear regression models were used to estimate the association between SAM in childhood and risk of hematological abnormalities.

**Results:**

Compared to the unexposed, the exposed had higher mean white blood cells (/μl) [+ 840 (179 to 1501), *p* = 0.013], neutrophils [+ 504 (83 to 925), *p* = 0.019] and platelets (*10^3^) [11.9 (8.1 to 17.9), *p* = 0.038] even after adjustment for food consumption in adulthood. No difference was observed in red blood cells (RBC), hemoglobin and erythrocytes parameters. With regard to the risk of hematological disorders, in contrast to the unexposed, exposed subjects had a risk of leukocytosis approximately three times higher [adjusted OR (95% CI): 2.98 (1.01 to 8.79), *p* = 0.048]. No difference was observed in terms of anemia, leukopenia, increased platelets and thrombocytopenia between the 2 groups.

**Conclusion:**

Adults with a history of SAM in childhood have hematological characteristics that would be markers associated with chronic low-grade inflammatory or infectious diseases in an environment with no nutritional transition. Larger cohort studies with bone marrow analyses could provide further understanding of the impact of SAM on the overall hematological profile in adult life.

## Background

Severe acute malnutrition (SAM) is a public health problem in low- and middle-income countries (LMICs). Its cost to the global economy was considered as high as US$3.5 trillion per year [1]. SAM currently affects 17 million children worldwide, 27% of whom live in Africa [1]. In addition, an estimated 2 million annual deaths are attributable to SAM [2]. Mortality is even higher when SAM combines with chronic malnutrition [3].

According to the World Health Organization (WHO), a child is considered to suffer from SAM when he or she has at least one among three criteria: Midd-Up Arm circumference (MUAC) <115 mm, weight to height Z-score (WHZ) < -3 Z-Score, and nutritional edema in the hands and/or feet and/or face [4].

In addition to mortality risk, SAM has serious adverse effects on many organs in survivors, especially when SAM occurs in early childhood [5, 6].

Studies in low-income countries (LICs) have shown that subjects with SAM remain at high risk of relapse and short-term mortality even after nutritional rehabilitation [7–9]. In addition, these subjects are at high risk of developing non-communicable diseases (NCDs) in adulthood [10–14]. Indeed, studies conducted in Uganda and Malawi (both LICs) showed that catching up after an episode of childhood wasting or stunting was positively associated with increased blood pressure (BP) in adolescence [15], and that prepubertal survivors of childhood SAM had an increased risk of acquiring modifiable risk factors for subsequent NCDs, although no clinical and biological markers could be found seven years after hospital discharge [16].

Among organs affected, the hematopoietic system is especially at risk [17–21]. As hematopoietic cells have a high proliferation index, they are more sensitive to changes in protein-energy reserves. As a rule, SAM leads to structural changes in the bone marrow microenvironment, as well as to functional alterations in mesenchymal cells and pluripotent cells, thus impairing hematopoiesis [22–25]. SAM in childhood causes changes in hematological tissues by impairing development and maturation of hematopoietic cells. This leads to anemia in addition to changes in reticulocyte response and erythroid activity, leukopenia (or hyperleukocytosis) and thrombocytopenia [25]. All these changes can lead to immunodeficiency [26]. SAM-related immunodeficiency may account for increased severity of common infectious diseases, including diarrhoea, pneumonia, measles, and malaria. As a result, children with SAM are more susceptible to severe acute life-threatening infections [3].

In the Democratic Republic of the Congo (DRC), malnutrition remains a major public health problem, with prevalence still above the critical threshold of 10% in some provinces [27–32]. Several studies have been conducted to explore the various specific aspects of SAM in DRC in general, and in South Kivu in particular [33–37]. Yet, none of these investigated the potential long-term deleterious effects of SAM during childhood on hematological parameters in adulthood.

Recently, in a cohort study (Lwiro Cohort) conducted in South Kivu, it was observed that adults with a history of SAM in childhood had a higher risk of developing a metabolic syndrome, visceral obesity and abnormal glucose homeostasis disorders than non-exposed community members, 11 to 30 years following nutritional rehabilitation [38]. Moreover, former SAM children had reduced human capital in adulthood [38]. Finally, it was observed that most deaths occurred within the five years following nutritional rehabilitation, and that most of these deaths were from to infectious diseases [39].

To date, very few studies have investigated the potential long-term consequences of SAM in early life on the overall hematological profile in adulthood living in LICs. Therefore, our study aimed to explore the association between SAM in childhood and hematological disorders in adulthood in a group of adults from the Lwiro cohort in the Eastern DRC evaluated 11 to 30 years after nutritional rehabilitation. This population from South Kivu has monotonous, undiversified, and low-quality diet, and nutritional transition has not yet taken place in this part of the DRC [32].

## Methods

### Study area, design and population

This was an observational follow-up study comparing adults with a history of previous hospital admission for SAM with community-living adult controls. Adults who were treated for SAM during childhood at Lwiro pediatric hospital (LPH) between 1988 and 2007, and still living in Miti-Murhesa and Katana Health zone (HZ) in 2018, were evaluated [38, 39]. A total of 1981 children were treated for SAM at LPH during the above period [39]. The nutritional status of study participants at the time of admission to hospital [37, 39, 40] was reassessed with the Emergency Nutrition Assessment (ENA) for SMART program (October 2007 version) based on WHO child growth’s standards [4]. Based on these standards, 1664 children were classified as having suffered from SAM [39]. The remainers were excluded from subsequent analyses. All children hospitalized for SAM were treated according to the guidelines used at that time [37]. For the initial group of follow up, 524 participants from the initial cohort still living in the two HZ were examined [38]. To assess long-term growth and health consequences of SAM, these survivors (SAM-exposed) were compared to 407 unexposed adult controls randomly-selected from the same community [38, 39].

The inclusion criteria for SAM-exposed individuals for this study were: to be at least 30 years of age, to have suffered for SAM before the age of 5 years, and to voluntarily agree to participate in the study.

In order to be included, unexposed controls should have the following characteristics: no hospital history of SAM, be of the same sex of the matched participants, live in the same community, and be less than 24 months older or younger than the exposed participants. Unexposed individuals were randomly selected by spinning a bottle at the exposed participant's home and enquiring door to door, starting from the nearest house towards which the bottle pointed [38, 39].

**Fig. 1:**
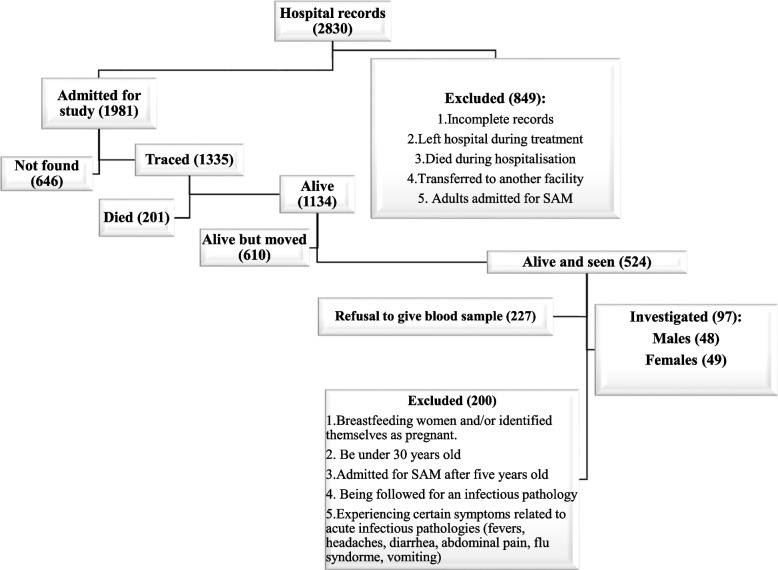
Recruitment of exposed group

On the basis of these criteria, 97 former SAM-sufferers’ adults were included and formed the exposed group, and matched to 97 non-exposed adult controls from potential participants (524 exposed and 407 unexposed) (Fig. 1).

The study exclusion criteria were: breastfeeding women and those who identified themselves as pregnant (more than 3 months of amenorrhea or with visible pregnancy), and being followed for an infectious pathology or experiencing certain symptoms related to acute infectious pathologies (fevers, headaches, diarrhea, abdominal pain, flu syndrome, vomiting) over the previous two weeks.

### Variables

#### Dependant variables

Our main outcomes of interest were hematological disorders including primarily anemia, leukopenia, leukocytosis, thrombocytopenia as well as increased platelets in adulthood, assessed by standard biological measures (blood cells count and erythrocytes parameters). We evaluated the associations between exposure to SAM during childhood and levels of red blood cells [red blood cells (RBC), hemoglobin (Hb) as well as erythrocyte constants: mean corpuscular volume (MCV), mean corpuscular hemoglobin concentration (MCHC) and mean corpuscular hemoglobin content (MCH)], white blood cells [white blood cells (WBC) and leukocyte count (neutrophils, lymphocytes and eosinophils)] and platelets.

Anemia was defined as Hb<13.0 g/dL in men and <12.0 g/dL in women; leukopenia as WBC <3500 WBC/μL, while leukocytosis was defined as WBC >11,000 WBC/μL. Thrombocytosis was defined as a platelets count < 150,000/μL, whereas increased platelets was defined as >450,000/μL.

BMI (Body Mass Index) was calculated as weight/height^2^ (in kg/m^2^) and categorized into four categories: <18.5 = underweight, 18.5 to 24.9 = normal, 25 to 29.9 = overweight and ≥ 30.0 = obese.

#### Independent variables

For hematological disorders and their different biological markers, the main exposure was a history of SAM in childhood. Lifestyle (food consumption) was added in the modelling as a potential confounder.

Food consumption frequency was evaluated using a diet diversity score (DDS) devised by the World Food Program (WFP) [29, 41, 42]. This score measures the dietary diversity of households, weighted by the frequency of consumption. The head of the household (often the mother) is asked how many days in the last seven days the household consumed any of the following 10 food groups (cereals, tubers, pulses, vegetables, fruit, meat/fish, milk/dairy, sugar, oil/fat, condiments). Next, the frequency of consumption of each food group was multiplied by its nutritional value, to yield a score per food group. Finally, the scores for each food group were added to obtain an overall score.

Based on this overall score, a subject was considered to have an inadequate, borderline or satisfactory diet if the score was between 0-28; 28.5-42 and >42, respectively [29, 42].

### Data collection

Data collection lasted approximately four months (August-November 2020) and was carried out in two stages by trained community health workers (CHWs). It was facilitated by the district authorities, the head nurses and community relays. The CHWs involved in data collection were the same people who had participated in the identification of subjects during the reconstitution of the exposure cohort [39].

The first step was a home visit. During these visits, the CHWs gave participants a questionnaire translated into Kiswahili, and took their anthropometric measurements. Participants were given an appointment at the nearest hospital for the second stage of data collection, within 48 hours. This second step consisted of obtaining a venous blood test by trained local nurses.

Unlike nurses and laboratory workers who were unaware of whether a participant was exposed or unexposed, CHWs were aware of this information. However, to avoid subjectivity and bias in the results, the context of the demographic data collection was presented as part of a routine exercise rather than as a comparison between exposed and unexposed.

The questionnaire included variables relating to the identity of the participant, anthropometric parameters and food consumption.

The anthropometric parameters considered were weight and standing height. Anthropometric measurements were performed according to WHO guidelines [4] and were quality controlled by having two team members independently measuring the anthropometric parameters. The encoded figure was the average of these two measures. If there was a difference of more than 300 g for weight, and/or 0.5 cm for standing height, a third measurement was taken, and the average of the two nearest measurements used.

Finally, 4 ml of blood was obtained from an antecubital venipuncture after 12 hours of fasting for the determination of the hemogram after counting on an automaton using standard calorimetric enzymatic methods (CYAN smart CY009, Brussels, Belgium) in the laboratory of the Bukavu provincial general reference hospital (tertiary level hospital).

### Statistical analysis

We used Stata software version 13.1 for statistical analyses. Categorical variables are summarized as frequency and proportion. Continuous variables are presented as mean and standard deviation (SD), as they all had symmetrical distribution.

Pearson's Chi^2^ or Fisher's exact test (if the conditions for applying Chi^2^ were not satisfied) were used for comparison of categorical variables and Student's t-test for comparison of means between groups.

Linear and logistic regression models were used for continuous variables [WBC, neutrophils, lymphocytes, RBCs, platelets, Hb and haematocrit (Hct), MCV, MCHC and MCHC] and categorical variables (anemia, thrombocytopenia, leukopenia, increased platelets and leukocytosis), respectively. The basic models included only the main exposure: SAM, thus providing a crude mean difference between exposed and unexposed for quantitative variables, and crude odds ratios (ORs) for categorical variables. The mean differences and ORs are shown with 95% confidence intervals (95% CIs).

To assess the potential confounding effect of adult dietary habits on the hematological profile, we added the food consumption score to the fitted model. To be included in the models, food consumption was dichotomized. We considered people with a satisfactory level of dietary diversity to be those with a fair food consumption score. The other categories were considered to have a "poor" level.

It should be noted that the conditions for the application of linear regression (normality, homoscedasticity and linearity) were checked by analysis of residuals and that the goodness-of-fit of the logistic model was verified by Hosmer-Lemehow’s test.

## Results

### Sociodemographic and nutritional characteristics of exposed and non-exposed subjects (Table 1)

**Table 1:** Demographic and nutritional characteristics of exposed and non-exposed subjects’ groups

	**Exposed**			**Non-exposed**		
	**N (97)**	**%**	**Mean** ± **SD**	**N (97)**	**%**	**Mean** ± **SD**
**Age (year)**			32.0 ± 2.8			32.0 ± 3.2
**Female**		50.5			50.5	
**Nutritional status**						
Weight (kg)			53.9 ± 7.1			55.4 ± 6.6
Height (cm)			156.2 ± 9.1			157.4 ± 9.4
**Body Mass Index** (kg/m^2^)			22.2 ± 3.3			22.4 ± 2.2
Underweight		6.2			2.1	
Normal		83.5			87.6	
Overweight/Obesity		10.3			10.3	
**Diet Diversity Score**			44.1 ± 10.0			46.8 ± 9.0
Insufficient		14.4			5.2	
Bordeline		36.1			34.0	
Satisfactory		49.5			60.8	

A total of 194 subjects were included in this study (97 exposed vs. 97 non-exposed). The mean age was 32 years in both groups with half of the subjects being female. The mean BMI was normal at around 22 kg/m^2^ with no significant difference between groups, although the exposed group had a higher proportion of underweight subjects. Finally, adults with a history of SAM tended to have a poor diet score compared to the unexposed.

### Difference in hematological parameters between exposed and non-exposed (Tables 2 and 3)

**Table 2:** Hematological profile in the 2 groups

	**Exposed (97)**		**Non-exposed (97)**		
	**%**	**Mean ± SD**	**%**	**Mean** ± **SD**	***p*** ** value** ^**1**^
*Figurative elements of blood					
**White Blood cells** (/ul)		7 207 ± 2 877		6 404 ± 1 575	0.017
Neutrophils (/ul)		3 688 ± 1 644		3 192 ± 1 279	0.02
Lymphocytes (/ul)		3 081 ± 1 179		2 940 ± 1 067	0.39
**Red BloodCells** (*10^3^/ul)		4 396.9 ± 549.1		4 495.8 ± 605.7	0.33
Haemoglobin (g/dl)		13.5 ± 1.6		13.7 ± 1.6	0.69
Haematocrit (%)		38.3 ± 4.1		38.9 ± 4.3	0.52
**Wintrobe’s Constant**					
VGM (fl.)		86.3 ± 4.6		85.5 ± 8.5	0.52
MCHC (g/dl)		35.3 ± 1.1		35.3 ± 1.2	0.81
MCH (pg)		30.5 ± 2.2		30.9 ± 4.7	0.44
**Platelets** (10^3^/ul)		241.4 ± 85.5		230.5 ± 77.8	0.021

^1^*p* value calculated using Student's t-test for means

**Table 3:** Adjusted difference in means (95%CI) of cellular Blood components in the 2 groups

	**Crude Difference (95% CI)**	***p*** ** value**	**Adjusted Difference (95% CI)** ^**a**^	***p*** ** value**
**White Blood Cells**	803 (146, 1459)	**0.017**	840 (17, 1501)	**0.013**
Neutrophils	496 (78, 913)	**0.02**	504 (83, 925)	**0.019**
Lymphocytes	141 (-180.1, 461.6)	0.39	131 (-18, 459)	0.41
Red blood cell*10^3^	- 98.9 (-262.7, 64.8)	0.33	- 98.0 (-263.1, 67.2)	0.24
Hemoglobin	-0.2 (-0.6, 0.2)	0.69	- 0.2 (-0.7, 0.3)	0.35
Hematocrit	-0.6 (-1.8, 0.6)	0.52	- 0.6 (-1.2, 0.6)	0.30
VGM	0.8 (-1.3, 2.6)	0.52	0.5 (-1.4,2.5)	0.59
MCHC	-0.4 (-1.5, 0.6)	0.81	-0.5 (-1.6, 0.5)	0.34
MCH	0.04 (-0.3, 0.4)	0.44	0.02 (-0.32, 0.35)	0.92
**Platelets** (10^3^)	10.9 (8.2, 16.2)	**0.021**	11.1 (8.1, 17.9)	**0.038**

^a^Difference with 95% CI (confidence interval) calculated by linear regression

Adjusted for diet diversity score dichotomized

In terms of hematological parameters, compared to unexposed, adults with a history of SAM had a significantly higher mean neutrophil count. In addition, they tended to have higher WBC, albeit still within normal range. However, no significant difference was observed in terms of mean WBC count. For the RBC line (overall mean RBC, Hb, Hct, anemia and erythrocytic constants), no significant differences were observed between the two groups. Finally, with regard to the platelet lineage, it was noted that SAM-exposed had a higher platelet count than the non-exposed, albeit still within normal range. However, there were no trends towards thrombocytopenia nor increased platelet counts. In the multivariate analysis (adjusted for diet diversity), it was observed that after adjustment for diet diversity score, former SAM adults had significantly higher WBC, neutrophil and platelet counts compared to the unexposed. The other parameters were similar in the two groups (RBCs and lymphocytes).

### Odds of hematological abnormalities (95% CI) between the 2 groups (Table 4)

**Table 4:** Adjusted effect of exposure on hematological abnormalities

	Exposed	Non exposed	**Crude OR (95% IC)** ^**a**^	*P* value	**Ajusted OR (95%IC)**	*P* value
Anemia	9.3	10.3	0.9 (0.3, 2.3)	0.81	0.9 (0.3, 2.4)	0.83
Leukocytosis	13.4	5.2	2.84 (0.9, 8.32)	**0.056**	2.98 (1.01, 8.79)	**0.048**
Thrombocytopenia	12.4	14.4	0.8 (0.4, 1.9)	0.67	0.8 (0.3,1.8)	0.62

^a^Odd ratio (OR) with 95% CI (confidence interval) calculated by logistic regression adjusted for diet diversity score dichotomized

Compared to the unexposed, it was observed that SAM-exposed had a 2.9-fold increased risk of leukocytosis, and this was significant even after adjustment for DDS. However, no difference in risk was observed in terms of anemia and thrombocytopenia between the two groups.

## Discussion

The aim of this work was to assess the association between childhood SAM and long-term hematological disorders through the analysis of blood counts of a group of adults with a history of SAM living in the eastern DRC.

This investigation was a sub-study of a larger survey (Lwiro Cohort Study) that attempts to contribute to understanding the long-term effects of SAM during childhood in LICs.

As regards the WBC line, our data suggest that former SAMs have a high risk of leukocytosis in adulthood and have high mean WBCs with a neutrophilic predominance. Indeed, studies of the hematological profile of children in the acute phase of SAM show almost systematic leukocytosis compared to non-SAM children [19–21, 43–47]. All these studies ascribed this leukocytosis as a marker of infections that malnourished children develop. These children have less activation of macrophagic cells linked to a low expression of GM-CSF (Granulocyte-Macrophage Colony-Stimulating Factor) and M-CSF (Macrophage Colony-Stimulating Factor), resulting in a stunted antibacterial immune response [25]. Indeed, it has been shown that SAM leads to atrophy of lymphoid organs (thymic and splenic), which blunts the antigenic response [5, 48, 49].

In addition, children with SAM develop environmental enteropathies with altered intestinal mucosa and barrier function that favor systemic microbial translocation from the gut with substantial bacterial sepsis [50]. A study carried out in Chile, in children recovering from SAM, revealed persistent deterioration of the intestinal mucosa although there was an improvement on electron microscopy of the border brush [51]. It has even been described that despite nutritional rehabilitation with normalization of anthropometric parameters, these children kept very high levels of inflammatory cytokines related to LPS (lipopolysaccharide) activation of gram-negative bacteria from the gastrointestinal tract, even when there were no clinical signs of gut infection [50–53].

The neutrophilia in this context would also be partly secondary to neutrophilic demargination and mobilization of the medullary neutrophils pool induced by calciprotein produced by injured endothelial cells in response to LPS [50]. Following correction of anthropometric parameters, there remained markers of damage to the intestinal microbiota and loss of integrity of the intestinal mucosa, with impaired micronutrients absorption [48, 50, 51]. This could also contribute to higher susceptibility to infections from the gut but also to risk of relapse in former malnourished children due to micronutrient deficiency. Hence these children in acute post-nutritional rehabilitation follow-up have a high mortality rate linked to infectious diseases [52, 8, 54]. This was suggested in a study conducted on the Lwiro cohort, in which the first cause of death during the first 5 years following nutritional rehabilitation were linked to infectious diseases, which were also responsible for a significant rate of relapse [39].

The main cohort of the Lwiro study showed a correlation between SAM and the occurrence of visceral obesity, metabolic syndrome, and carbohydrate homeostasis disorder in adulthood [38]. These diseases are among the chronic NCDs that are frequently associated with a chronic subclinical low-grade inflammation component [55]. The metabolic profile of children with SAM differed from that of healthy children and did not normalize after growth resumption, as observed in several studies conducted in LICs [51, 55, 56]. Apart from the infectious context, joint responses between neutrophils and platelets during chronic inflammatory diseases could contribute to the combination of elevated neutrophil count and platelet counts reported here.

Angèle Gros and coll. demonstrated that platelets and neutrophils are involved in the development of chronic inflammatory diseases [57, 58]. Several experimental and human models, described in a literature review published in January 2022, suggest platelet receptors involvement in platelet and leukocyte recruitment, but also in vascular permeability, via formation of platelet-neutrophil or platelet-macrophage complexes, present in chronic inflammatory diseases such as rheumatoid arthritis, or in ischemic stroke and atherosclerosis in general. These complexes are linked to the expression of platelet receptors as well as to cytokines secreted by mononuclear cells, mainly neutrophils [59]. In addition, atherosclerotic patients have neutrophil-platelet complexes that promote inflammatory response based on neutrophil migration and activation [60]. Thus, a trend to elevated neutrophilia and platelets could be viewed in the Lwiro cohort as markers of inflammation in the context of metabolic syndrome and central obesity, two chronic non-communicable inflammatory diseases.

In terms of the RBCs line (RBC, Hb, Wintrobe’s constant and risk of anemia), no statistically significant difference was observed between exposed and non-exposed. This lack of difference could result from the benefit of nutritional rehabilitation, which would have provided all necessary nutrients for subsequent RBC production, such as amino-acids, iron and vitamins.

This observation was highlighted in studies conducted in Palestine and Burkina faso on the evaluation of the impact of a humanitarian project to combat SAM [61–63]. In these studies, the prevalence of anemia in a group of malnourished people was almost halved following food supplementation [61–63].

Finally, a tendency for elevated platelets in exposed cases could be explained in part by inflammation due to a prevalence of infections but also to the acquisition of a metabolic syndrome phenotype [38].

Our study has some obvious limitations:

First, there is a survival bias: only those who survived to adulthood (of 30 years) and were still present in local villages two to three decades after the SAM episode were studied. Nevertheless, there is no obvious reason to believe that the association between nutritional status of children hospitalized for SAM and hematological profile would have been different among those lost to follow-up, as the characteristics at hospital admission did not differ between the lost and traced subjects [39].

Secondly, the sample size of our study was not very large. Some differences could have become statistically significant with a larger sample size.

Thirdly, we did not have data on bone marrow, spleen and liver status nor on other markers of inflammation and infection. Indeed, changes in these parameters could have contributed to the hematological disturbances independently of SAM.

Fourthly, we did not have serum iron status and transferrin saturation data, which are related to platelet aggregation parameters, nor had measures of micronutrients involved in hematopoiesis.

Fifth, it is questionable whether all non-exposed people recruited were healthy. Although they did not experience kwashiorkor nor were treated for SAM, it is possible that some of them had some degree of malnutrition related to poor socio-economic conditions of the region, which did not result in hospitalization. This permanent unfavorable situation in which the two groups evolved may have contributed substantially to attenuating possible differences in many biological markers under study. Sixthly, the design of our study was unable to establish causality and it is therefore difficult to separate mechanisms due to SAM *per se* from mechanisms due to the persistent effects of the early childhood environment or the persistence of a precarious environment in which the subject continued to live on the observed abnormalities.

## Conclusion

Adults with a history of SAM in childhood have hematological characteristics that would be markers associated with chronic low-grade inflammatory or infectious diseases in an environmental context without nutritional transition. Larger cohort studies including bone marrow analyses could provide better understanding of the impact of SAM on the overall hematological profile in adult life. Researchers and health policy makers should invest more in understanding the long-term effects of SAM to combat its prevalence as a preventive health measure

## Data Availability

All data generated or analyzed during this study are included in this published article.

## Abbreviations

BMI: Body Mass Index
BP: Blood pressure
CHWs: Community health workers
DDS: Diet diversity score
DRC: Democratic Republic of the Congo
HZ: Health Zone
LICs: Low-income countries
LMICs: Low- and middle-income countries
LPH: Lwiro Pediatric Hospital
LPS: Lipopolysaccharide
MCH: Mean corpuscular hemoglobin content
MCHC: Mean corpuscular hemoglobin concentration
MCV: Mean corpuscular volume
NCDs: Non-communicable diseases
OR: Odd ratio
RBC: Red blood cells
SAM: Severe acute malnutrition
WBC: White blood cells
WHO: World Health Organization
